# Evolutionary and Developmental Biology Provide Insights Into the Regeneration of Organ of Corti Hair Cells

**DOI:** 10.3389/fncel.2018.00252

**Published:** 2018-08-08

**Authors:** Karen L. Elliott, Bernd Fritzsch, Jeremy S. Duncan

**Affiliations:** ^1^Department of Biology, University of Iowa, Iowa City, IA, United States; ^2^Department of Biological Sciences, Western Michigan University, Kalamazoo, MI, United States

**Keywords:** organ of Corti evolution, hair cell planar cell polarity, hair cell kinocilium loss, MET channel mutation effects, PCP effects on sensory neurons

## Abstract

We review the evolution and development of organ of Corti hair cells with a focus on their molecular differences from vestibular hair cells. Such information is needed to therapeutically guide organ of Corti hair cell development in flat epithelia and generate the correct arrangement of different hair cell types, orientation of stereocilia, and the delayed loss of the kinocilium that are all essential for hearing, while avoiding driving hair cells toward a vestibular fate. Highlighting the differences from vestibular organs and defining what is known about the regulation of these differences will help focus future research directions toward successful restoration of an organ of Corti following long-term hair cell loss.

## Introduction

All vertebrate hair cells (HCs) have stereocilia organized in a staircase pattern, displaying distinct apical polarities for stimuli to open mechanoelectrical transduction channels (METs) permitting endolymphatic potassium to enter the HCs and change their resting potential (Hudspeth, 2005; Reichenbach and Hudspeth, 2014). All vertebrates have vestibular-like HCs (vHCs) in ears (Lewis et al., 1985; Desai et al., 2005) and, if present, in lateral line mechanosensors (Fritzsch and López-Schier, 2014; Chagnaud et al., 2017; Nicolson, 2017). However, only mammals possess specialized HCs within mammalian-specific organ of Corti (OC) that differ from vHCs (Fritzsch et al., 2013; Manley, 2017). This distinctiveness is most obvious in their stereocilia organization and the absence of kinocilia (Schwander et al., 2010; Fritzsch and Elliott, 2017). There is also a profound difference in the radial distribution of HCs and supporting cells in the OC (Held, 1902; Jahan et al., 2015a) that is unlike the checkerboard distribution of HCs and supporting cells in the vestibular organs (Desai et al., 2005; Jahan et al., 2015a; Burns and Stone, 2017). In addition to HC morphology and distribution differences, there is a unique innervation by afferent (Rubel and Fritzsch, 2002; Dabdoub et al., 2016) and efferent neurons (Simmons et al., 2011; Sienknecht et al., 2014; Maison et al., 2016) and unique OHC contractility (Okoruwa et al., 2008; He et al., 2014).

Both vHCs and OC-HCs die over time, leading to late onset vestibular and hearing dysfunction (Rauch et al., 2001; Fattal et al., 2018). One approach to combat age-related HC loss is to generate new HCs in their place. Non-mammalian vertebrates are able to regenerate HCs throughout life, and this is also possible for vestibular organs of mice (Bucks et al., 2017). In contrast, mammalian OC-HCs are unable to regenerate beyond an early age (White et al., 2006), even if additional molecular measures convert remaining supporting cells into HC-like cells (McLean et al., 2016; Walters et al., 2017). The reasons for the inability of OC-HCs to regenerate may be related, in part, to the unique cellular structure of the OC, the unique organization of stereocilia on the apical surface of OC-HCs, and the late embryonic/neonatal loss of the kinocilium.

**FIGURE 1:**
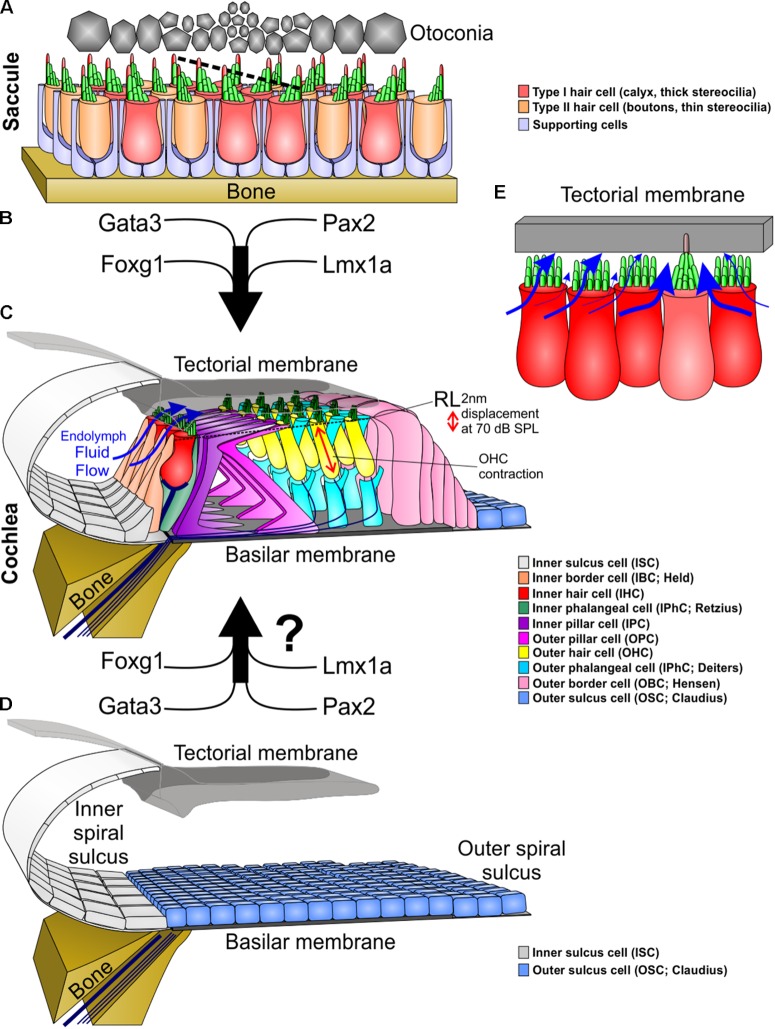
Molecular and morphological differences between vestibular and cochlear hair cells. **(A)** Type I and type II vestibular hair cells, arranged in a checkerboard pattern with supporting cells, form opposing polarities in the saccule at a line of polarity reversal (dotted line, center). **(B)** The organ of Corti evolved out of an outgrowth of the saccule into the lagenar duct and requires expression of several genes to form organ of Corti-specific hair cell subtypes and distribution. **(C)** The organ of Corti has a unique organization of hair cells and supporting cells to form one row of inner hair cells and three rows of outer hair cells separated by two rows of supporting cells (inner and outer pillar cells). Following a downward deflection of the reticular lamina (RL) by 2 nm at 70 dB sound pressure level (SPL; more at higher sound intensities), endolymph from the inner spiral sulcus flows over the stereocilia of inner hair cells (blue arrows) and into the subtectorial space that is in addition modified by the prestin mediated outer hair cell contractility to amplify local movements. **(D)** We hypothesize that recapitulation of the genes involved in the evolution and development of the organ of Corti might be needed to transform the flat epithelium, consisting of BMP4 positive outer sulcus (Claudius) cells, following hair cell loss to restore the organ of Corti. **(E)** Substitution of an inner hair cell by a vestibular type hair cell would alter the fluid flow mechanics. Instead of the endolymph primarily moving over the top of stereocilia, it would move around the vestibular stereocilia bundle, causing little movement of stereocilia. In addition, stereocilia movement is reduced by the kinocilium being embedded in the tectorial membrane. This kinocilia in the tectorial membrane would also affect the vibration of the tectorial membrane relative to the basilar membrane. Thickness of blue arrows denotes relative fluid flow.

Thus far, all attempts to generate HCs in the dish have resulted in only the formation of vHCs (Liu et al., 2016; Koehler et al., 2017), that are unlikely to function in the OC. While some direct conversions have resulted in OC-like HC types, most attempts generated abnormal HCs that either could not function or have limited viability (Yang et al., 2012; Walters et al., 2017). This review provides an overview of our current understanding of specific aspects of OC-HC versus vHC morphological and molecular development. We evaluate available developmental and evolutionary data to better molecularly understand the necessary steps that can transform vHCs into those of the OC (**Figure 1**) and transform a vestibular organ with unsymmetrically distributed HC types into a precisely organized OC with two distinctly distributed types of structurally and functional unique OC-HCs (Jahan et al., 2015a).

## Molecular Developmental Evolutionary Considerations

In the vestibular organs, the sensory epithelia are mostly organized as a repeating mosaic of supporting cells and HCs. In contrast, the OC contains several types of highly specialized supporting cells and the HCs are organized in a very stereotyped single row of inner hair cells (IHC) and 3 rows of outer hair cells (OHC) surrounded by variable numbers of supporting cells (Lim, 1986) (**Figure 1**). It has been hypothesized that during evolution, new sensory epithelia evolved by the expansion and subsequent partitioning of preexisting epithelia during development (Duncan and Fritzsch, 2012a; Mann et al., 2017). This also most likely occurred in the formation of the OC by an expansion of the saccular sensory epithelia into the lagenar recess that eventually developed into the cochlea (Fritzsch et al., 2013; Basch et al., 2016). As stated above, the attempts to regenerate HCs results in the formation of vestibular like HCs, perhaps due to the OC’s evolutionary vestibular past (Yang et al., 2012). Deciphering developmental genetic differences between the vestibular epithelia and the OC amounts to understanding the changes in gene regulatory networks that originally drove the evolution of the OC. This information will not only advance the understanding of how a novel sensory modality evolved, but will benefit hearing restoration by regenerating auditory HCs instead of driving cells toward a vestibular fate (Jahan et al., 2018).

Unlike vestibular epithelia, the OC grows through extension into the elongating cochlear duct (Burns et al., 2012; Driver et al., 2017). This growth due to the precise regulation of proliferation, convergent extension, and differentiation results in the unique cellular arrangement of the OC. We have just begun to understand how this can be interpreted as the evolution of transcription factors (for example: Foxg1, Lmx1a, N-Myc, and Neurod1) that expand the OC driving its growth from the ductus reuniens toward the apical end (Montcouquiol et al., 2003; Fritzsch et al., 2013; Driver et al., 2017). Mutations of these genes result in a shortened OC that has multiple rows of both IHCs and OHCs, may show unusual distribution of OC-HC (Neurod1), or develops OC-vHC mixes (Lmx1a). Thus, the extension of the cochlear duct and with it, the convergent extension of the OC precursor cells, must promote the formation of the four rows of HCs. The loss of *Foxg1* results in 16 or more rows of HCs within this shortened OC (Pauley et al., 2006), more extensive compared to loss of Neurog1 (Ma et al., 2000). These cochleae resemble monotreme mammals that naturally have multiple rows of HCs in the apex of a shortened cochlear duct (Ladhams and Pickles, 1996; Fritzsch et al., 2013). It is thus conceivable that studying the development of the monotreme OC would reveal the factors necessary for convergent extension (Montcouquiol et al., 2003) and rearrangement of multiple rows of HCs into four rows; with *Foxg1* being likely involved. How the differential control of proliferation and differentiation affects convergent extension processes that are involved in the outgrowth of the OC by intercalation of precursor cells is not fully known (Driver et al., 2017).

Unlike the vestibular system, the IHCs and OHCs of the OC are segregated (**Figure 1**). While the comingling of type I and type II HCs within the vestibular epithelia does not compromise their function, the segregation of IHCs and OHCs is crucial for OC functionality. The OHC cells provide signal amplification (He et al., 2014; Xia et al., 2018) via the tectorial membrane (Russell et al., 2007; Dewey et al., 2018) creating a differential flow of fluid exciting the IHCs. This function requires at a minimum that IHCs lose their kinocilia in order for their stereocilia to move freely from the inner spiral sulcus to the subtectorial space, and back (**Figure 1C**). To achieve this, IHCs have thick stereocilia and are immediately adjacent to each other to maximally obstruct the fluid flow from the sub-tectorial space to the inner spiral sulcus (Richter et al., 2007) (**Figure 1C**). Since the reticular lamina is only displaced by approximately 2 nm at 70 dB sound pressure level (Ren et al., 2016), it is essential to have this maximal obstruction of fluid flow so that limited movements of endolymph at the tallest stereocilia causes enough displacement to stimulate IHCs (Hudspeth, 2005; Reichenbach and Hudspeth, 2014). Embedding a kinocilium into the overlying tectorial membrane and reshaping IHC stereocilia like vHC bundles would hamper this IHC function (**Figure 1E**). In contrast, presence of a kinocilium and embedding it into the tectorial membrane is fully compatible with basilar papilla mediated sound sensing in other vertebrates (Manley, 2017). Beyond morphological descriptions of the loss of kinocilia (Kimura et al., 1964; Lindeman et al., 1971) nothing is known about the molecular cues underlying this delayed loss with retention of the basal body that appears unique to OC HCs. A possibility would be co-opting tubulin disassembly mechanisms from cell division to rapidly disassemble the kinocilia.

These physiological and phylogenetic considerations suggest that OC-HC development and evolution was a stepwise transformation: this achieved the right diameter of stereocilia with the right number and overall organization in the right cell type in conjunction with the prestin mediated OC amplifier (Okoruwa et al., 2008). It also ensured the delayed loss of the kinocilium that is initially necessary for orientation of the HC and its ability to detect diffusible signals such as Shh (Corbit et al., 2005; Bok et al., 2013; Sienknecht et al., 2014). Indeed, the natural late loss of the primary cilium (aka kinocilium) in OC-HCs does not result in aberrant development that occurs when most epithelial cells lose their kinocilium. However, when certain kinocilia proteins are mutated (Jones et al., 2008) or there is a developmental reduction of kinocilia (Delmaghani et al., 2016) there is an effect on the normal development of cochlear HCs, indicating that the kinocilia is necessary early in development and its loss is also crucial for HC function in the OC. Minimally we need to understand this process to the extent that we can form a kinocilia to generate OC-HCs, but then force the loss of the kinocilium in order for HCs to properly function within the OC. It stands to reason that both of these process are embedded downstream of several unique transcription factors (**Figure 1**) that either selectively affect HCs of the OC if mutated such as Gata3 (Karis et al., 2001; Duncan and Fritzsch, 2013), Pax2 (Torres et al., 1996; Bouchard et al., 2010) or transform part of the OC into a vestibular HC carrying organ such as Lmx1a (Nichols et al., 2008).

## Differences Between Vestibular and Cochlear Stereocilia and Their Mechanotransduction Channels

The mammalian mechanosensory channel is in part formed by the transmembrane proteins Tmc1/2 (Pan et al., 2013). Knocking out Tmc1 or 2 differentially affects vHCs and OC-HCs. This may be related to differences in the spatiotemporal expression profile of these proteins between the vestibular and cochlear systems (Imtiaz et al., 2016; Shibata et al., 2016). While Tmc1 null mice are deaf as adults, vestibular function is not impaired. In contrast, Tmc2 null mice have normal hearing and vestibular function. Tmc2 is postnatally downregulated in OC-HCs, whereas vHCs retain Tmc2, suggesting Tmc2 can compensate for loss of Tmc1 in vHCs whereas there is a specific requirement of OC-HCs for Tmc1 (Kawashima et al., 2015). Even more profound are differences between vHCs and OC-HCs regarding the likely MET associated protein, CIB2 (Giese et al., 2017; Vélez-Ortega and Frolenkov, 2017; Vélez-Ortega et al., 2017). While *Cib2* null mice progressively lose OC-HCs, there is no change in vHCs. Moreover, HC loss starts in the IHCs, progressing from base to apex, much like in Cdc42 mutants that affect the actin assembly in the stereocilia (Ueyama et al., 2014). Furthermore, OC-HCs are more susceptible to degeneration after embryonic ablation of afferents whereas vHCs are more robust (Kersigo and Fritzsch, 2015), suggesting that differences in physiological properties include differential sensitivity to various components of the mechanotransduction system. While our understanding of the relative necessity of parts of the mechanosensory channel between the vHCs and OC-HCs is greatly increasing, there is a lack of understanding of both why these differences are necessary for specialization of these different HCs and the gene regulatory networks that drive these differences during development (Booth et al., 2018; Ellwanger et al., 2018).

Differential effects of stereocilia homeostasis extend beyond induced cell death after mutations of Cdc42 (Ueyama et al., 2014) and also include differential effects of stereocilia length after mutations in epidermal growth factor receptor pathway substrate 8 [Eps8 (Tavazzani et al., 2016)]: Such mutants are deaf but show no obvious vestibular phenotype, reinforcing the perception of differences between OC-HCs and vHCs. In part, these differences are related to the presence of kinocilia in vHCs that allow mechanical stimuli to be transmitted to the shorter stereocilia, implying that in the vestibular system, kinocilia are part of the mechanotransduction apparatus allowing the tallest stereocilia to participate in mechanotransduction. Furthermore, vHCs have a normal MET current in these mutants, which is substantially altered in OC-HCs (Tavazzani et al., 2016). Moreover, the much-shortened IHC stereocilia essentially disable the function of these HCs as hydrodynamic sensors that monitor fluid flow between the subtectorial space (between the tectorial membrane and the reticular lamina) and the inner spiral sulcus (**Figure 1**). These data on stereocilia geometry on hearing (Tavazzani et al., 2016) add to the complexity of the cellular assembly of the OC (Jahan et al., 2015a) and its emerging regulation by changes such as replacing Atoh1 by Neurog1 (Jahan et al., 2015b), changing timing of cell cycle exit and Atoh1 regulation (Kopecky et al., 2013) or altering a range of diffusible factors that cooperate with delta-notch signaling to fine tune that pattern (Groves and Fekete, 2012, 2017; Munnamalai and Fekete, 2016).

## Planar Cell Polarity in Inner Ear Neurosensory Development

**FIGURE 2:**
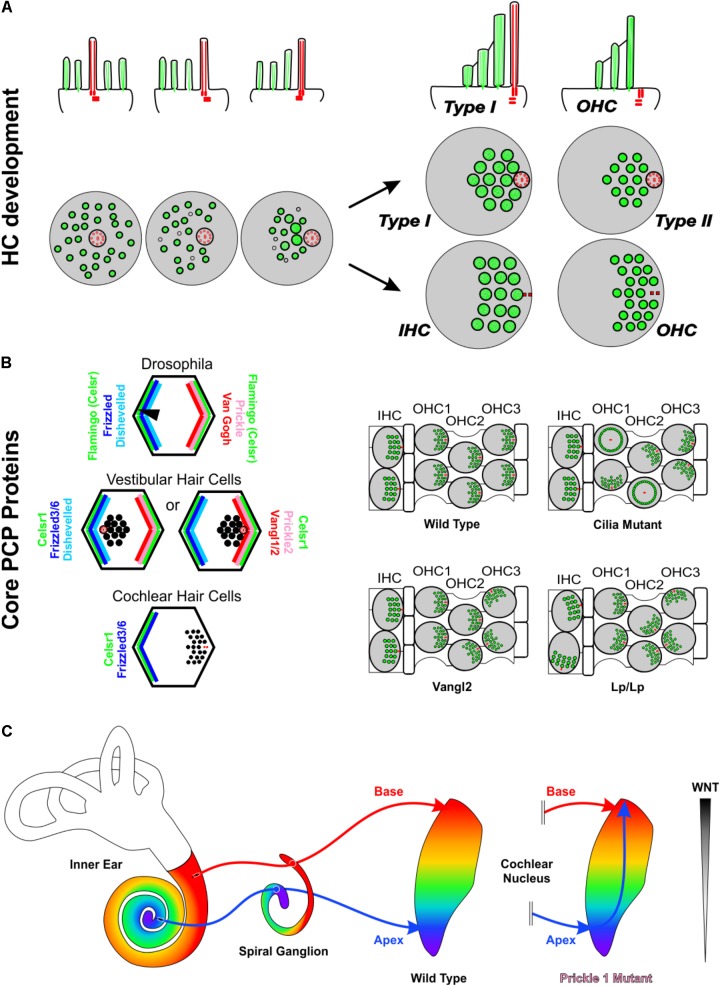
Asymmetries in Inner ear development. **(A)** Development of stereocilia and kinocilia asymmetric localization on the apical portion of hair cells. The top row is a side view while the bottom row is a top down view of hair cell kinocilia in red and microvillistereocilia in green. As development progresses, the kinocilium migrates into an off-center position devoid of microvilli. The microvilli trailing the kinocilium grow in length and thickness and eventually develop into stereocilia. As the kinocilium reaches its asymmetric position, the stereocilia have formed their typical staircase arrangement whereas microvilli have disappeared. Differential development generates four general types of hair cells with distinctive stereocilia diameter and distribution. In the organ of Corti, hair cells eventually lose their kinocilia. The side view shows a Type I vestibular hair cell and a cochlear outer hair cell. **(B)** The distribution of PCP proteins in Drosophila, vestibular hair cells, and cochlear hair cells and resulting orientations of hair bristle/stereocilia (black). In the fly and in vestibular hair cells, Prickle (Prickle 2) and Van Gogh reside on one side of the cell, Frizzled and Disheveled on the other and Flamingo/Celsr1 is distributed on both sides of the cell. In vestibular hair cells, while the distribution of PCP proteins remains constant, stereocilia orient in opposing polarities at the line of polarity reversal. In cochlear hair cells, Frizzled3/6 and Celsr1 are expressed on one side, opposite the stereocilia. Data on mutations in PCP genes in the vertebrate suggest that the loss of individual PCP proteins differentially affects hair cells within an epithelium and hair cells between epithelia. **(C)** In Prickle1 mutants, there is an overlap of branches of apical afferents (blue arrow) with basal afferents (red arrow), in contrast to the maintenance of tonotopic projections in wild type mice. Based on this phenotype, we hypothesize that for spiral ganglion afferent segregation the diffusible Wnts factors released from the dorsal hindbrain (gray gradient) play a role. Abbreviations: HC, hair cell; IHC, Inner hair cell; OHC, outer hair cell; Vangl1/2, Van Gogh-like 1/2; Lp, Looptail.

All HCs, no matter their sub-type, initiate their development with the kinocilium in the center of their apical surface surrounded by microvilli. As development progresses, this kinocilium translocates toward one side of the HC and the microvilli specialize as stereocilia (**Figure 2A**). As they specialize, the stereocilia closest to the kinocilium end up being the tallest while those further away are shorter, resulting in a staircase pattern. This directionality toward one side of the cell gives the HC an orientation, known as polarity (Hudspeth, 2005). Comparative analyses show that the developmental progression of HC polarization resembles that of the evolution from single-celled ancestor of all metazoans to vertebrate mechanosensory cells. In fact, it has been suggested that transformation of the concentric arrangement of apical structures of the choanoflagellate to a more polarized distribution like that of the vertebrate HC is mediated by genes involved in polarity and HC development (Duncan and Fritzsch, 2012a; Fritzsch and Elliott, 2017). This polarization is coordinated, resulting in all HCs of a particular region to be oriented in the same direction. Across most vertebrate taxa, the orientation pattern of the utricle is conserved (Lewis et al., 1985) with two regions of HCs with opposing orientation patterns, abutting at the line of polarity reversal. In contrast, the saccule has 2–6 regions with distinct orientation patterns depending on the species (Lewis et al., 1985; Ladich and Schulz-Mirbach, 2016). In species with two orientation patterns, the two regions of HCs are oriented away from each other. Both the utricle and saccule polarity patterns are substantially different from the mammalian OC where all HCs are oriented with their tallest stereocilia located abneurally. A single polarity for all vHCs is also found in canal cristae but polarity of horizontal crista is different from that of posterior and anterior canal crista (Lewis et al., 1985) possibly due to their late addition (Maklad et al., 2010). The OC-HC single orientation is especially striking as the OC evolved as an outgrowth of the saccule (Duncan and Fritzsch, 2012b). Whether the OC is only the outgrowth of a small region of the saccule with a single orientation or evolved from an intermediate form that contained two orientations of HCs remains unknown but has important ramifications in replacement of cochlear HCs with uniform polarity. The necessity of this information in regeneration is highlighted by the fact that the proper orientation of cochlear HCs is necessary for their function, and when experimentally inducing HCs in the greater epithelial ridge, these HCs lack coordinated orientation (Kelly et al., 2012). Below we provide some insights into these processes.

Planar cell polarity (PCP) mechanisms control the development of polarity in individual cells and coordinate the alignment of neighboring cells across epithelia. Mutations in PCP proteins do not affect all HCs equally. For example, in the absence of Vangl2 it is primarily the third row of OHCs that display orientation defects while the other rows appear more normal (**Figure 2B**). Similarly, in the utricle only the HCs of the striola are affected whereas the rest of the epithelia remain close to normal (Yin et al., 2012). Interestingly, where the utricle and cochleae are different is when these mice are allowed to develop further. In this circumstance, many of these orientation defects within the cochlea are refined closer to a normal orientation, whereas the orientation defects within the vestibular utricle remain (Copley et al., 2013). Loss of only Vangl1 does not seem to have any effect on OC HCs (Song et al., 2010). However, the Vangl2 looptail mutation has been shown to also restrict the function of Vangl1 and is a severe mutation that results in the misorientation of all four rows of HCs in the OC (Montcouquiol et al., 2003; Yin et al., 2012) (**Figure 2B**). Like Vangl2, loss of Celsr1 seems to also only affect the 3^rd^ row of outer HCs within the OC and the striola of the utricle (Duncan et al., 2017). Another family of PCP proteins within the OC is Fzd3/6. A double mutation in both Fzd3 and Fzd6 results in a phenotype that is in stark contrast with Vangl2 or Celsr1. In the Fzd3/6 mutant, the inner row of HCs has a severe defect in orientation whereas the third rows are less affected (Wang et al., 2006). These data on canonical PCP proteins is consistent with the mutation of Wnt5a, a ligand of Fzd receptors (Sienknecht et al., 2014). In Wnt5a mutants, HCs show altered orientations (Qian et al., 2007), and several mouse mutants possess sensory epithelia that remained fused and display intermediate PCP orientations (Duncan and Fritzsch, 2012b). Another PCP protein, Prickle 2, affects only the utricle (Deans et al., 2007).

The various subtypes of inner ear HCs are connected to cochlear and vestibular nuclei through cochlear and vestibular afferents, respectively (Rubel and Fritzsch, 2002; Dabdoub et al., 2016) (**Figure 2C**), and correct navigation to cochlear nuclei can occur even in the absence of central targets (Elliott et al., 2017). Transplantation studies (Elliott et al., 2013, 2015) suggest that inner ear afferents navigate to the correct dorsoventral column in the hindbrain using diffusible molecular cues from the hindbrain (Fritzsch and Elliott, 2017). Since it is thought that HCs and their associated neurons arose from a single neurosensory cell through gene duplication and differential association (Duncan and Fritzsch, 2012a; Fritzsch and Elliott, 2017), it is not surprising that PCP proteins have been shown to play a role in afferent navigation (Tao et al., 2012; Okerlund et al., 2016) in addition to their role in HC polarity. Interestingly, mice mutant for the PCP protein, Prickle 1, had no effect on OC-HC polarity, but rather on spiral ganglion afferent pathfinding (Yang et al., 2017). These mice displayed an overlap of apical afferents with basal afferents within cochlear nuclei (**Figure 2C**) (Yang et al., 2017). Given the role of several other PCP proteins in axon guidance in other neuronal systems (Zou, 2004; Hua et al., 2014; Qu et al., 2014; Chai et al., 2015; Wang et al., 2017), it is likely that additional PCP proteins play also a role in inner ear afferent navigation.

In summary, mammalian inner ears contain several HC subtypes that vary in their morphology and organization within and between different sensory epithelia and species. Current attempts to generate new HCs have thus far yielded only vHC types; however, these vHCs will not function in the OC due to presence of kinocilia and stereocilia organization and will thus not be able to restore their lost hearing at the needed sensitivity. Understanding the differential molecular evolution and development leading to HC heterogeneity may lend insights into the unique processes that result in the formation of the correct HC subtype in the right position with the proper polarity essential for function, including connection of HCs to their central targets by afferents.

## Author Contributions

KE contributed to the conceptualization, wrote part of the paper, contributed to **Figures 1, 2** and edited all of the text and figures. BF contributed to the conceptualization, wrote the introduction and contributed to **Figure 1**. JD conceptualized the paper, wrote the body of the paper and developed **Figure 2**.

## Conflict of Interest Statement

The authors declare that the research was conducted in the absence of any commercial or financial relationships that could be construed as a potential conflict of interest. The reviewer JM and the handling Editor declared their shared affiliation.
